# MicroRNA expression patterns in the malignant progression of gliomas and a 5-microRNA signature for prognosis

**DOI:** 10.18632/oncotarget.2679

**Published:** 2014-11-04

**Authors:** Wei Yan, Rui Li, Yanwei Liu, Pei Yang, Zheng Wang, Chuanbao Zhang, Zhaoshi Bao, Wei Zhang, Yongping You, Tao Jiang

**Affiliations:** ^1^ Beijing Neurosurgical Institute, Capital Medical University, Beijing, PR China; ^2^ Department of Neurosurgery, First Affiliated Hospital of Nanjing Medical University, Nanjing, PR China; ^3^ Beijing Institute for Brain Disorders Brain Tumor Center, Beijing, PR China; ^4^ Department of Neurosurgery, Beijing Tiantan Hospital, Capital Medical University, Beijing, PR China

**Keywords:** Anaplastic Gliomas, Secondary Glioblastomas, Proneural Glioblastomas, MicroRNAs, MiR-105-767 cluster, TCGA

## Abstract

MicroRNAs (miRNAs) are directly involved in the progression in various cancers. To date, no systematic researches have been performed on the expression pattern of miRNA during progression from low grade gliomas to anaplastic gliomas or secondary glioblastomas and those prognostic miRNAs in anaplastic gliomas and secondary glioblastomas. In the present study, high-throughput microarrays were used to measure miRNA expression levels in 116 samples in the different progression stages of glioma. We found that miRNA expression pattern totally altered when low grade gliomas progressed to anaplastic gliomas or secondary glioblastomas. However, anaplastic gliomas and secondary glioblastomas have similar expression pattern in miRNA level. Furthermore, we developed a five-miRNA signature (two protective miRNAs-miR-767-5p, miR-105; three risky miRNAs: miR-584, miR-296-5p and miR-196a) that could identify patients with a high risk of unfavorable outcome in anaplastic gliomas regardless of histology type. It should be highlighted that the five-miRNA signature can also identify patients who had a high risk of unfavorable outcome in secondary and TCGA Proneural glioblastomas, but not Neural, Classical and Mesenchymal glioblastomas. Taken together, our results demonstrate that miRNA expression patterns in the malignant progression of gliomas and a novel prognostic classifier, the five-miRNA signature, serve as a prognostic marker for patient risk stratification in anaplastic gliomas, Secondary and Proneural glioblastomas.

## INTRODUCTION

MicroRNAs (miRNAs) belong to a recently discovered class of small, non-coding RNA molecules that regulate the expression of multiple target genes and multiple cellular processes including cell differentiation, stem cell maintenance, and epithelial–mesenchymal transition.[1, 2] The abnormal expression of miRNAs is a common feature of cancers and can be caused by different mechanisms such as amplification/deletion, chromosomal rearrangements, and epigenetic regulation.[3, 4] Depending on the genes targeted, miRNAs can act as either oncogenes or tumor suppressors.[5-8] MiRNAs show characteristic expression signatures in various cancers and can profoundly affect cancer cell behavior[9, 10]. Low grade gliomas inherently tend to locally recur and spontaneously progress to anaplastic gliomas and eventually secondary glioblastomas.[11] However, to date, the miRNA expression patterns in the malignant progression of gliomas have not been investigated systematically.

In this study, we present the results of miRNA expression patterns from 116 glioma samples by microarray analysis. We found that miRNA expression pattern totally altered when low grade gliomas progressed to anaplastic gliomas or secondary glioblastomas. However, anaplastic gliomas and secondary glioblastomas have similar expression pattern in miRNA level. Meanwhile, survival analysis revealed that a five-miRNA signature (two protective miRNAs-miR-767-5p, miR-105; three risky miRNAs: miR-584, miR-296-5p and miR-196a) that could identify patients with a high risk of unfavorable outcome in anaplastic gliomas, secondary and TCGA Proneural glioblastomas, but not Neural, Classical and Mesenchymal glioblastomas.

## RESULTS

### Whole genome miRNA profiling reveals miRNA expression patterns in the malignant progression of gliomas

**Figure 1:**
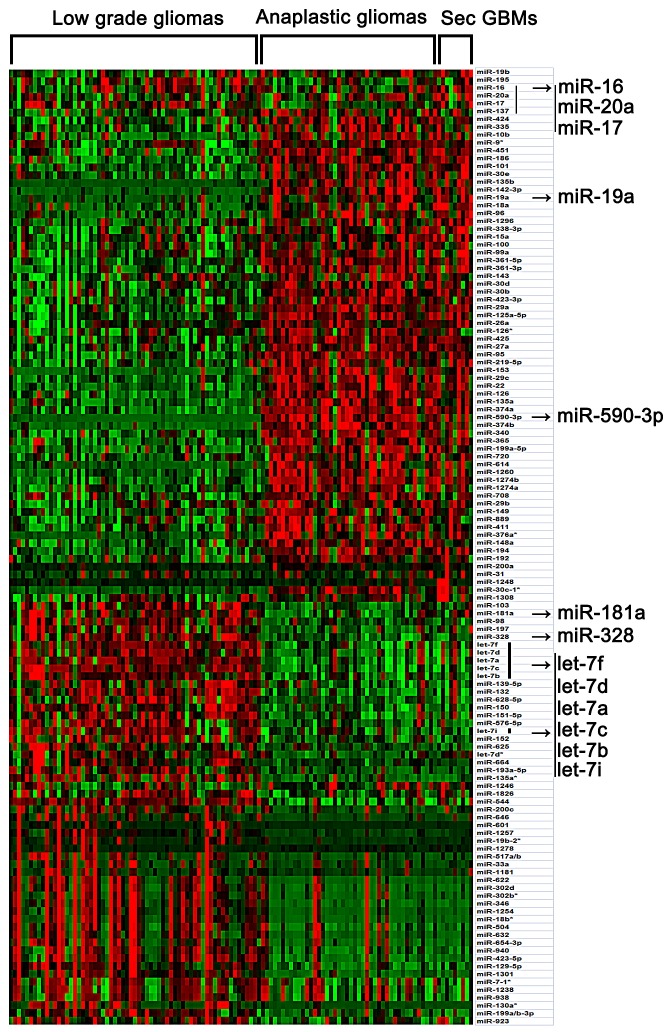
Differential expressed miRNAs among low grade, anaplastic gliomas and secondary glioblastomas (SAM: Fold change > 1.5, Q value < 1%) Total 116 samples were ordered from low grade glioma to secondary glioblastomas, and differential expressed miRNAs were clustered. Arrows indicated genes that were discussed in the text.

In the present study, SAM was performed to compare the differential expressed miRNAs among low grade, anaplastic gliomas and secondary glioblastomas in a pairwise manner (Supplementary Figure 1). Only those miRNAs (SAM: Fold change > 1.5, Q value < 1%) were taken as significant miRNAs. Then samples were ordered from low grade glioma to secondary glioblastomas, and significant genes were clustered (Figure 1). As shown in Figure 1 and Supplementary Figure 1, the miRNA expression pattern of secondary glioblastomas resembles that of anaplastic gliomas very much. It is very possible that the main alteration of miRNAs occurred during malignant progression from low-grade glioma to anaplastic gliomas or secondary glioblastomas, but not anaplastic gliomas to secondary glioblastomas. In the present study, we observed aberrant expression of several well-characterized tumorigenesis-related miRNAs (such as miR-16, miR-17, miR-19a, miR-20a, miR-328, miR-181a and let-7 family members), which also showed the same increased or reduced expression upon progression, as well as a number of miRNAs whose function is currently unknown (such as miR-590-3p). In addition, we also validated the expression of miR-590-3p in an independent validation cohort [Supplementary Figure 2].

### Identification of the five-miRNA signature and its association with survival in anaplastic gliomas

**Figure 2:**
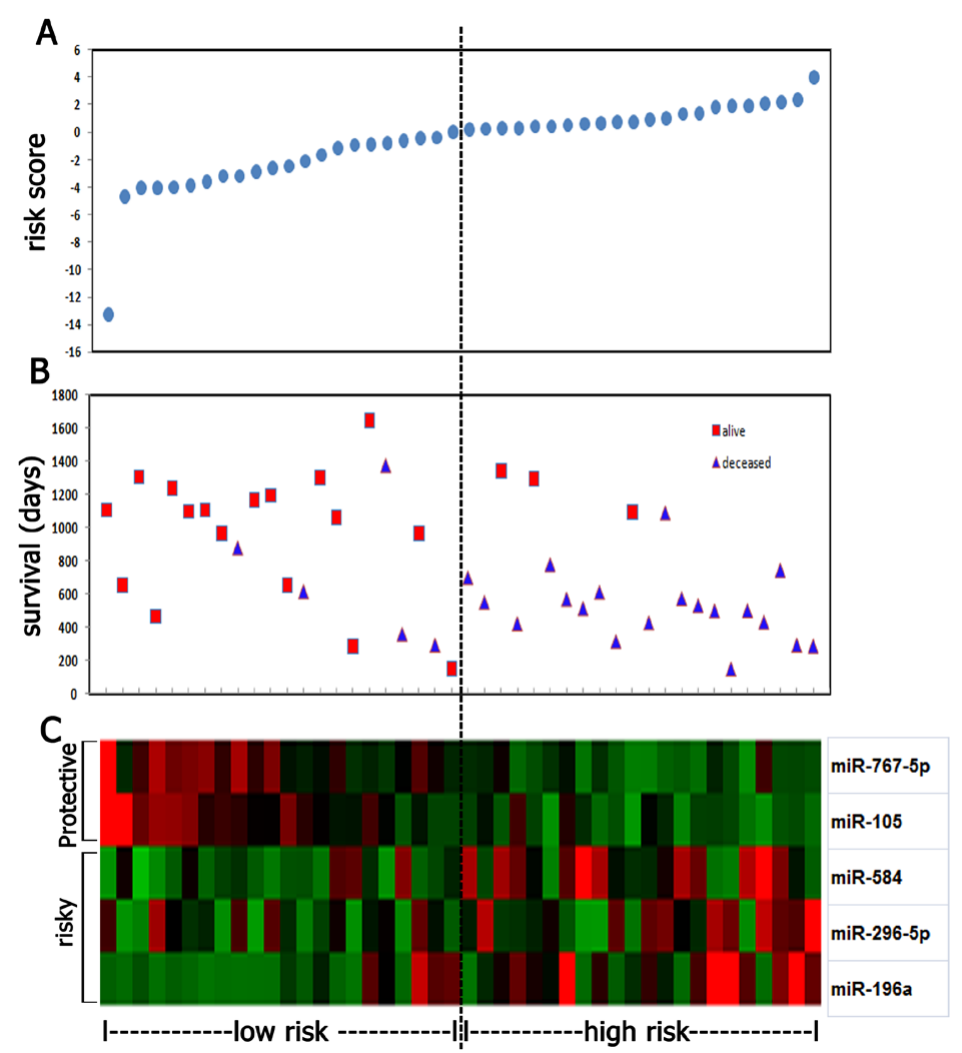
Five miRNA risk-score analysis of anaplastic gliomas (n=44) (A) The prognostic miRNA signature risk-score distribution. (B) Patients' survival status and time. (C) Color-gram of miRNA expression profiles of GBM patients; rows represent risky and protective miRNAs, and columns represent patients. The black dotted line represents the miRNA signature cutoff dividing patients into low-risk and high-risk groups.

**Figure 3:**
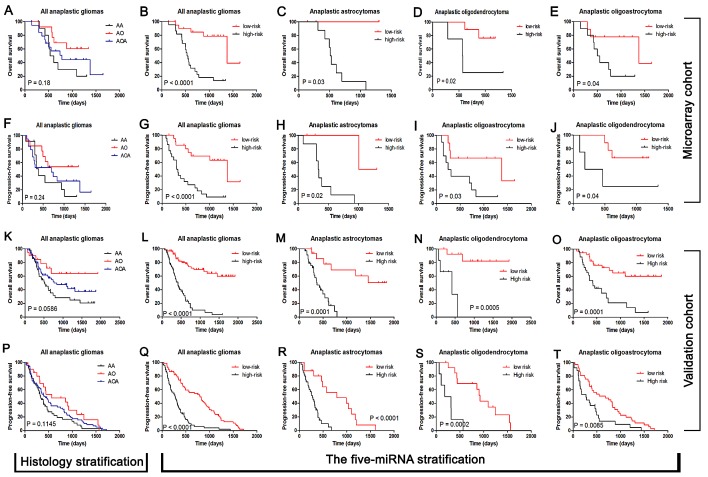
The five-miRNA signature was tightly associated with prognosis in both Microarray cohort (n=44) and Validation cohort (n=134) independent of histology type Histology types could stratify the anaplastic gliomas into different prognostic subpopulation although the P value is not enough small in both Microarray (A and F).and Validation cohort (K and P). The five-miRNA signature was could stratify the all anaplastic gliomas into two distinct prognostic subpopulation in both Microarray (B and G).and Validation cohort (L and Q). In each histological subtype of anaplastic gliomas (astrocytomas, oligodendrocytomas and oligoastrocytomas), the five-miRNA signature could also be taken as a stable method used for prognosis stratification in both Microarray (C, D, E, H, I and J) and Validation cohort (M, N, O, R, S and T).

Following data filtering, described in the “Materials and Methods”, the remaining 629 miRNAs were analyzed by BRB array tools using the permutation test method. We identified five miRNAs (miR-767-5p, miR-105, miR-584, miR-296-5p and miR-196a) that are significantly associated with overall survival (Permutation P < 0.01; Figure 2). The significant miRNAs that formed the signature were of two types - risky and protective. Risky miRNAs were defined as those that had hazard ratio for death greater than 1. Protective miRNAs were defined as those that had hazard ratio for death less than 1. Using this definition, we found 2 protective miRNAs and 3 risky miRNAs. Of note, 2 protective miRNAs: miR-105 and miR-767-5p are located in the same miRNA cluster. We then used the five miRNAs identified to construct a signature by the risk-score method.[15] As shown in Figure 3A-J, the five-miRNA signature risk score was calculated for each of the 44 patients in the Microarray cohort and then was used to divide them into a high-risk group and a low-risk group in all anaplastic gliomas, astrocytomas, oligodendrocytomas and oligoastrocytomas. Furthermore, the prognostic value of the five-miRNA signature was validated using real-time qRT-PCR in an independent cohort consisting of 134 samples (Figure 3K-T).

### The five-miRNA signature predict the clinical outcome of secondary and TCGA Proneural glioblastomas

**Figure 4:**
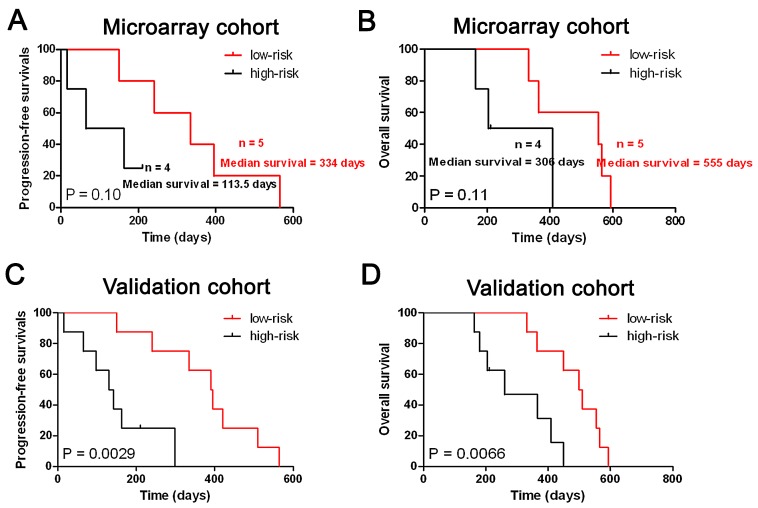
The five-miRNA signature was tightly associated with prognosis in secondary glioblastomas The five-miRNA signature was could stratify the all secondary glioblastomas into two distinct prognostic subpopulation in both Microarray (A and B).and Validation cohort (C and D).

In the present study, secondary glioblastomas have similar miRNA expression pattern with anaplastic gliomas. So we further evaluated whether the five-miRNA signature could identify patients with a high risk of unfavorable outcome in secondary glioblastomas. As shown in Figure 4, the five-miRNA signature was also associated with the prognosis of secondary glioblastomas.

**Figure 5:**
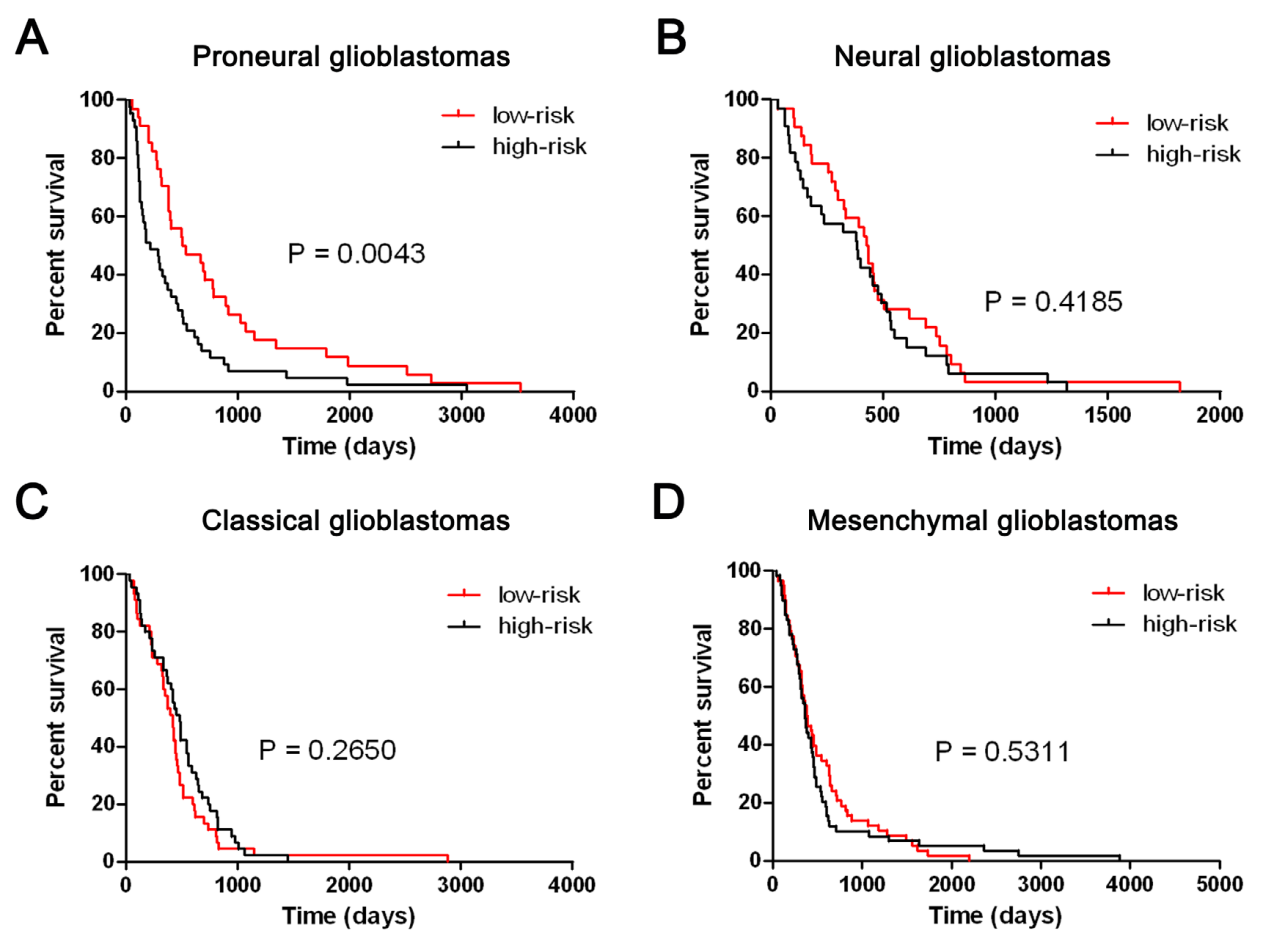
The five-miRNA signature could predict the clinical outcome of TCGA Proneural glioblastomas The five-miRNA signature was could stratify TCGA Proneural glioblastomas into two distinct prognostic subpopulation (A). However, the five-miRNA signature could not predict the clinical outcome in Neural, Classical and Mesenchymal glioblastomas (B, C and D).

To validate the five-miRNA signature in TCGA glioblastomas, paired miRNA and mRNA profiling data (level3) was downloaded from TCGA data portal. Total 491 TCGA glioblastoma samples were included in our study. We first annotated the TCGA glioblastoma samples with TCGA classification system based on mRNA expression. After removing the patients whose survival time were lesser than 30 days, Total 349 patients (77 Proneural, 65 Neural, 90 Classical and 117 Mesenchymal glioblastomas) who had survival information were for further analysis. As shown in Figure 5, the five-miRNA signature could identify patients with a high risk of unfavorable outcome in Proneural glioblastomas (P = 0.0043). However, the five-miRNA signature could not predict the clinical outcome in Neural, Classical and Mesenchymal glioblastomas (P = 0.4185, 0.265, and 0.5311, respectively).

## DISCUSSION

MiRNAs show characteristic expression signatures in various cancers and can profoundly affect cancer cell behavior.[16] Low grade gliomas inherently tend to locally recur and spontaneously progress to anaplastic gliomas and eventually secondary glioblastomas.[11] However, to date, the miRNA expression patterns in the malignant progression of gliomas have not been investigated systematically. In the present study, we analyzed the whole-genome miRNA profiles 116 samples in the different progression stages of glioma and found that miRNA expression pattern totally altered when low grade gliomas progressed to anaplastic gliomas or secondary glioblastomas. However, anaplastic gliomas and secondary glioblastomas have similar expression pattern in miRNA level. In addition, several progression associated miRNAs in previous reports (miR-16, miR-17, miR-19a, miR-20a, miR-328, miR-181a and let-7 family members) also showed the same increased or reduced expression upon progression in our study.[11, 17] Our observations indicated that miRNA may play a critical role during progression from low grade gliomas to anaplastic gliomas or secondary glioblastomas and not contribute to the malignant progression from anaplastic gliomas to secondary glioblastomas.

Anaplastic gliomas are classified by the WHO as grade 3 malignant tumors and include anaplastic astrocytomas, anaplastic oligodendrogliomas and anaplastic oligoastrocytomas.[18] It has previously been shown that miRNA expression patterns could be used as prognostic indicators in various cancers.[19, 20] Recently, *Srinivasan et al.* reported that a ten-miRNA expression signature predicts survival in glioblastoma.[15] However, to date, no prognostic markers based on miRNA profiling have been identified in anaplastic gliomas. In the present study, we developed a five-miRNA signature (two protective miRNAs-miR-767-5p, miR-105; three risky miRNAs: miR-584, miR-296-5p and miR-196a) that could identify patients with a high risk of unfavorable outcome in anaplastic gliomas regardless of histology type. Of them, only miR-196a was previously reported to be negatively correlated with overall survival in glioblastomas.[21] It should be highlighted that two protective miRNAs (miR-767-5p and miR-105) localized in the same miRNA cluster. Our observations showed a novel prognostic classifier, the five-miRNA signature, in anaplastic gliomas and revealed an important role for the miR-105-767 cluster in the pathogenesis and tumor biology of anaplastic gliomas for the first time. In addition, we applied the five-miRNA signature in secondary and TCGA glioblastomas. We found that the five-miRNA signature can also identify patients who had a high risk of unfavorable outcome in secondary and TCGA Proneural glioblastomas, but not Neural, Classical and Mesenchymal glioblastomas. The molecular and clinical characteristics of Proneural samples in primary glioblastomas resemble secondary glioblastoma and may progress from lower grade gliomas thought without clinical or histological evidence of a less malignant precursor lesion. Thus, we postulated that Proneural glioblastomas might have similar miRNA expression pattern with anaplastic gliomas and secondary glioblastomas and undergo potential malignant progression from low grade gliomas without a previous operation.

In summary, our results demonstrate that miRNA may play a critical role during progression from low grade gliomas to anaplastic gliomas or secondary glioblastomas and not contribute to the malignant progression from anaplastic gliomas to secondary glioblastomas. We further provide a novel prognostic classifier, the five-miRNA signature, serve as a prognostic marker for patient risk stratification in anaplastic gliomas, Secondary and Proneural glioblastomas.

## PATIENTS AND METHODS

### Patients and samples

All glioma samples included in our study were from the Chinese Glioma Genome Atlas (CGGA). The patients underwent surgical resection between January 2006 and December 2009. Patients were eligible for the study if the diagnosis of glioma was established histologically according to the 2007 WHO classification. Tumor tissue samples were obtained by surgical resection before treatment with radiation and chemotherapy. This study was approved by the institutional review boards of the hospitals, and written informed consent was obtained from all patients.

### RNA extraction, Whole genome miRNA profiling and Real-time qRT-PCR

RNA extraction, Whole genome miRNA profiling and Real-time qRT-PCR were performed as Ref.[12] Briefly, total RNA from frozen tumor tissues was extracted using the mirVana miRNA Isolation kit (Ambion, Austin, TX, USA) according to the manufacturer's protocol. Then, 200 ng of total RNA was polyadenylated and then converted into cDNA using a biotin-labeled Oligo dT primer with a universal PCR sequence. After cDNA synthesis, miRNA were individually interrogated using specific oligonucleotides. A single miRNA-specific Oligo (MSO) was designed against each mature miRNA sequence, and miRNA-specific primers were extended using DNA polymerase. Universal primers were used to amplify the cDNA templates, and the primer complimentary to the array was fluorescently labeled. Finally, the labeled, single-stranded PCR products were hybridized to the Human v2.0 miRNA Expression BeadChip (Illumina, Inc., San Diego, CA, USA) containing 1,146 human miRNAs (97% coverage of the miRBase 12.0 database). Real-time qRT-PCR was performed using a standard TaqMan PCR kit procedure on a LightCycler 480 real-time PCR system (Roche). All of the primers and probes for the TaqMan microRNA assays were purchased from Applied Biosystems. Real-time RT-PCR was carried out according to the manufacturer's recommendation, and the relative expression was calculated using the comparative Ct method.

### Data filtering and statistical analysis

Median absolute deviation (MAD) was calculated using MATLAB software. After filtering the probes showing the lowest variable expression (MAD < 0.05), the remaining 629 miRNAs were used for the following analysis. We applied significance analysis of microarrays (SAM) to identify differential expressed miRNAs in low grade, anaplastic gliomas and secondary glioblastomas in a pairwise manner. Student's t-test was used to determine significant differences. Cox proportional hazard regression analyses were performed using the BRB array tools in the Microarray cohort. Using the permutation test method, with 10000 permutations, we found 5 miRNAs were strongly correlated with survival (p < 0.01). The significant miRNAs were divided into risky and protective types. Risky miRNAs were defined as miRNAs with a hazard ratio for death greater than 1. In contrast, protective miRNAs were defined based on a hazard ratio for death less than 1. Using these 5 significant miRNAs, a risk-score formula for survival prediction was constructed according to a linear combination of the expression level of the miRNA, weighted by the regression coefficient from the univariate Cox regression analyses.[13-15] According to this model, patients having high risk scores are expected to have poor survival outcomes as compared to patients having low risk scores. The risk scores are calculated as follows: Risk score = (−5.04 × expression of hsa-miR-767-5p) + (6.5 × expression of hsa-miR-296-5p) + (0.76 × expression of hsa-miR-584) + (−3.14 × expression of hsa-miR-105) + (0.9 × expression of hsa-miR-196a). We used the 50th percentile risk score as the cut-off, since this divided the Microarray samples into two groups having different survival times with highest significance. Kaplan-Meier survival analysis was used to estimate the survival distributions, and the log-rank test was used to assess the statistical significance between stratified survival groups using GraphPad Prism 5.0 statistical software. Prediction analysis of microarrays was used to subtype annotation. All data are presented as the means ± SE. A two-sided P value of < 0.05 was regarded as significant.

## SUPPLEMENTARY MATERIAL AND FIGURES




