# Supplementary material for: MicroRNA expression patterns in the malignant progression of gliomas and a 5-microRNA signature for prognosis
Source: Oncotarget. 2014 Nov 4;5(24):12908–15. doi: 10.18632/oncotarget.2679 (PMC4350338; doi:10.18632/oncotarget.2679)
Supplement: Supplementary file 1 [file oncotarget-05-12908-s001.pdf]

MicroRNA expression patterns in the malignant progression of gliomas and a 5-microRNA signature for prognosis

Supplementary Material

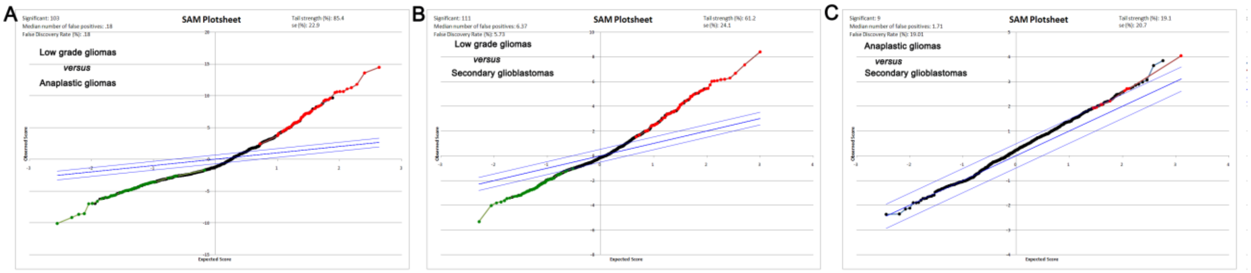

Supplementary Figure 1: SAM plots for comparing differential expressed miRNAs between (A) low grade gliomas and anaplastic gliomas, (B) low grade gliomas and secondary glioblastomas, (C) anaplastic gliomas and secondary glioblastomas.

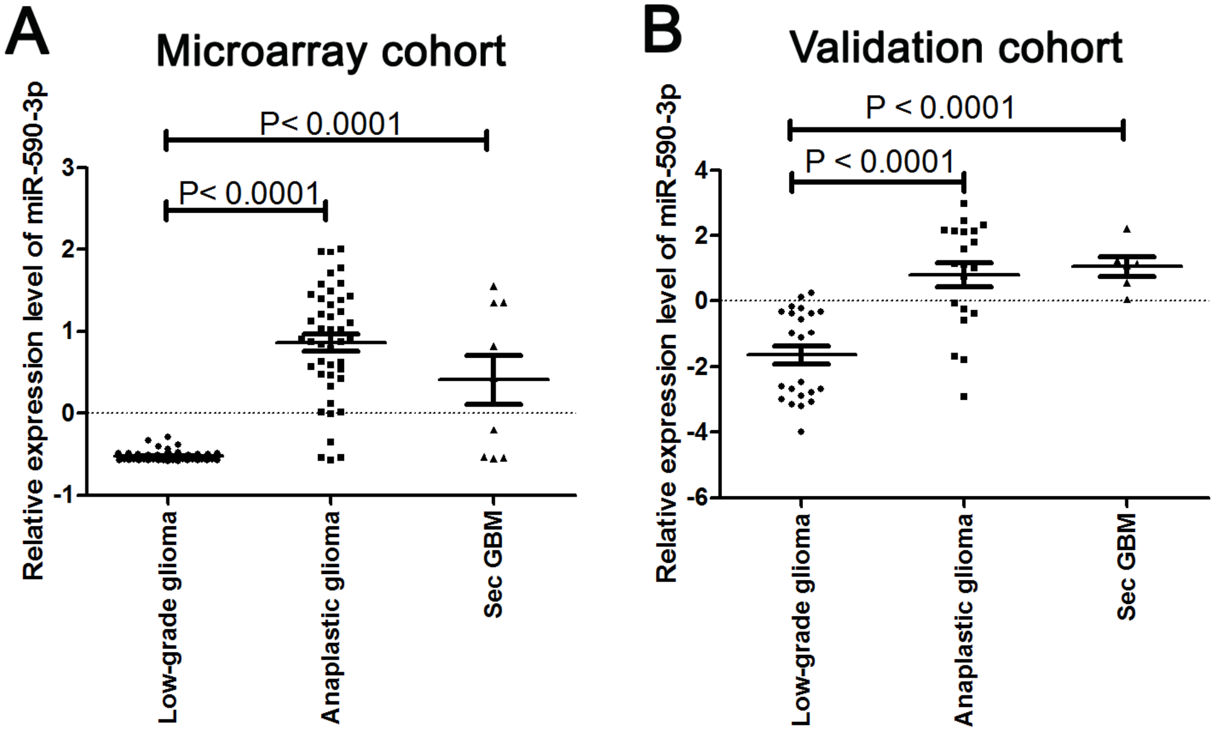

Supplementary Figure 2: MiRNA-590-3p expression levels in both Microarray (A) and Validation cohort (B).
